# Vascular feature identification in actinic keratosis grades I-III using dynamic optical coherence tomography with automated, quantitative analysis

**DOI:** 10.1007/s00403-024-03022-z

**Published:** 2024-06-15

**Authors:** Gabriella Fredman, Stine R. Wiegell, Merete Haedersdal, Gavrielle R. Untracht

**Affiliations:** 1 Department of Dermatology, Copenhagen University Hospital, Bispebjerg and Frederiksberg, Copenhagen, NV 2400 Denmark; 2 Department of Clinical Medicine, Faculty of Health and Medical Science, University of Copenhagen, Copenhagen, Denmark; 3 Department of Health Technology, Technical University of Denmark, Kongens Lyngby, 2800 Denmark

**Keywords:** Dynamic optical coherence tomography, OCTAVA, Actinic keratosis, Skin microvasculature, Angiography, Non-invasive imaging

## Abstract

Clinical grading of actinic keratosis (AK) is based on skin surface features, while subclinical alterations are not taken into consideration. Dynamic optical coherence tomography (D-OCT) enables quantification of the skin´s vasculature, potentially helpful to improve the link between clinical and subclinical features. We aimed to compare microvascular characteristics across AK grades using D-OCT with automated vascular analysis. This explorative study examined AK and photodamaged skin (PD) on the face or scalp. AKs were clinically graded according to the Olsen Classification scheme before D-OCT assessment. Using an open-source software tool, the OCT angiographic analyzer (OCTAVA), we quantified vascular network features, including *total* and *mean vessel length*, *mean vessel diameter*, *vessel area density* (VAD), *branchpoint density* (BD), and *mean tortuosity* from enface maximum intensity projection images. Additionally, we performed subregional analyses on selected scans to overcome challenges associated with imaging through hyperkeratosis (each lesion group; *n* = 18). Our study included 45 patients with a total of 205 AKs; 93 grade I lesions, 65 grade II, 47 grade III and 89 areas with PD skin. We found that all AK grades were more extensively vascularized relative to PD, as shown by greater total vessel length and VAD (*p* ≤ 0.009). Moreover, AKs displayed a disorganized vascular network, with higher BD in AK I-II (*p* < 0.001), and mean tortuosity in AK II-III (*p* ≤ 0.001) than in PD. Vascularization also increased with AK grade, showing significantly greater total vessel length in AK III than AK I (*p* = 0.029). Microvascular quantification of AK unveiled subclinical, quantitative differences among AK grades I-III and PD skin. D-OCT-based microvascular assessment may serve as a supplement to clinical AK grading, potentially raising perspectives to improve management strategies.

## Introduction

Actinic keratosis (AK) is an epidermal, premalignant lesion predominantly located in sun-exposed skin, with a high prevalence among middle-aged and elderly individuals [1, 2]. Multiple, solitary AKs usually co-exist with subclinical lesions within continuous areas of photodamaged skin, known as field-cancerization [1–3]. Field cancerization holds significant clinical and therapeutic implications, since this concept emphasizes that AK do not adhere to discrete, clearly defined stages [4]. Recognizing this complex nature of AK underscores the need for effective treatment strategies to enhance patient management.

In clinical practice, evaluating AK involves several challenges because of the heterogenous course of individual lesions and their variable response to treatment. If left untreated, AK most often become chronic, but individual lesions may either regress or evolve into squamous cell carcinoma (SCC), although at a relatively low rate [1–3]. The clinical evaluation of AK is traditionally based on grading scales that usually rely on surface features. Most commonly applied is the 3-step Olsen Classification Scheme, which categorizes AK according to their clinical thickness [5]. This scheme has limited utility in both clinical practice and research trials since it falls short in identifying underlying subclinical changes [4, 6].

To address these limitations, recent advancements in non-invasive imaging have been fundamental. In particular, dynamic optical coherence tomography (D-OCT) excels in its ability to provide high-resolution three-dimensional images of the skin´s microvascular network to depths of up to 500 μm using near-infrared light [7, 8]. This technology not only improves detection and diagnosis of AK but also deepens our insights into vascular involvement in disease development [8]. Until now, D-OCT research has focused on qualitative analysis of vessel morphologies, a method prone to inter-observer variability. This limitation underscores the need for automated approaches to harness the full potential of D-OCT in understanding and treating AK.

In efforts to broaden D-OCT´s capability to characterize the skin microvasculature and standardize evaluation, the OCT angiographic vascular analyzer (OCTAVA) was recently developed [9]. OCTAVA is an open-source software tool that provides quantitative information about the microvasculature in D-OCT images. This software enables users to extract and analyze multiple parameters related to the vascular network’s structure and function. The opportunity to apply a large number of parameters for microvascular characterization using OCTAVA may bring possibilities to detect subclinical variations across AK grades and relative to PD skin. Such a comprehensive approach holds strong prospects to identify biomarkers that improve the link between clinical classification and underlying subclinical alterations. In our explorative study, we aimed to identify microvascular features associated with the clinical AK grades I-III using OCTAVA.

## Methods and materials

### Design and ethical considerations

This explorative study was undertaken at the Department of Dermatology, Copenhagen University Hospital, Bispebjerg and Frederiksberg in Copenhagen and HudCenter Privathospitalet Mølholm in Vejle, Denmark. Patients with AK and PD skin were included from October 2021 - September 2022 as part of a larger investigation of AK patients. The study was approved by the Ethics Committee of Region Hovedstaden (78,842) and registered in EudraCT (2021-0015860-21). The unit for Good Clinical Practice monitored the study, which was conducted in accordance with the Declaration of Helsinki. Before study initiation, all patients signed an informed consent.

### Study set-up

The study´s inclusion criteria were patients over the age of ≥ 18 years with clinically visible AKs on the face or scalp. Exclusion criteria were any AK therapy up to 3 months before inclusion, or any skin disease other than AK inside the test sites.

Following inclusion, AKs were marked and numbered on a transparent film before evaluation. In the clinical evaluation, the same board-certified dermatologist (SW) graded AK by thickness using the Olsen Classification Scheme from I: mild (slightly palpable, better felt than seen), II: moderate (moderately thick, easily felt and seen), and III: severe (very thick or obvious, hyperkeratotic) [5, 10]. Evaluation with D-OCT included at least one AK grade I, II, III and adjacent PD skin within each test site.

### Dynamic OCT – image acquisition

This study used a commercially available D-OCT scanner (Vivosight Dx, Michelson Diagnostics, Kent, UK). The D-OCT scanner has a center wavelength of 1305 nm, lateral resolution of < 7.5 μm, axial resolution of < 5 μm, and a field-of-view of 6 × 6 mm [7, 11]. Structural OCT images of the skin are captured up to a depth of 1.5 mm, and the in-built Vivosight software automatically generates the vascular D-OCT images. Each D-OCT en-face image is displayed overlayed on the structural OCT en-face scan. Vessels are visualized up to a maximum depth of ≤ 500 μm. Imaging below this depth is limited by the interference of speckle and low signal to noise ratio in deeper parts of the skin [7, 12, 13]. In our study, volumetric image acquisition was performed with 250-B scans (in the x-z plane).

### Automated vessel analysis using OCTAVA

From each D-OCT scan, maximum intensity projection (MIP) images were generated using MATLAB 2023a (Mathworks, inc., Natick, MA, USA.) centered at a depth of 300 μm from the skin surface and comprising a thickness of 100 μm. Quantitative vascular parameters were extracted from the MIP images using the OCTAVA software (v2, MATLAB App version) [9]. Briefly, OCTAVA generates metrics by binarizing and skeletonizing images to identify the connectivity of the vascular network. Nodes and segment lengths are calculated using graph analysis, and vessel diameters are calculated using a Euclidian distance transform [9]. All metrics are saved to an Excel file for further analysis. For this study, all images were processed in batch processing mode with the same settings. The fuzzy means segmentation was used, and the Frangi filter was applied with a minimum kernel size 1 and a maximum kernel size 8. These settings were optimized for D-OCT images of skin in a previous study [9].

In our study, the vascular parameters assessed using OCTAVA are described in Table 1. OCTAVA quantifies *total vessel length* and *vessel area density* (VAD) to assess the extent of the vascular network [9]. In addition, the software provides measurements of the average size of blood vessels, including *mean vessel length* and *mean vessel diameter*, crucial for evaluating the vascular network´s capacity. Additional parameters relate to the vascular network´s organization. Of these, branchpoint density (BD) serves as an indicator for vessel’s connectivity within the network, reflecting the frequency of vessel branching. Additionally, *mean tortuosity* relates to the extent of twisting and turning of blood vessels, which may influence tissue perfusion.

**Table 1:** Overview and description of quantitative vessel parameters assessed using OCTAVA

Metric	Unit	Description
Total length	µm	Cumulative length of all vessels across the entire en-face image
Mean length	µm	Mean length of individual vessels in each en-face image
Mean diameter	µm	Mean diameter of individual vessels in each en-face image
Vessel area density	%	Proportion of en-face image occupied by perfused vessels
Branchpoint density	nodes/mm^2	Number of branchpoints within the vascular network
Mean tortuosity	NA	Twisting of vessels, evaluated using the arc length-over-cord ratio

NA; Not applicable

Thick hyperkeratosis frequently interfered with D-OCT imaging of AK III, which prevented a reliable vessel quantification in these lesions. To enable a representative comparison of vascular differences across all AK grades and PD, we analyzed selected subregions free from image artifacts caused by hyperkeratosis in a subset of AK I-III and PD skin (each group; *n* = 18) using the grid of 9 squares provided by OCTAVA. The selection of subregion from each lesion group was based on specific characteristics identified through qualitative evaluation of vessels in D-OCT scans of AK I-III and PD skin, as described in our previous work (unpublished). In this prior study, we systematically evaluated various vessel shapes, vessel patterns, and vessel directions characteristic of each AK grade. Briefly, these analyzes revealed a structured vessel pattern in AK I lesions, resembling that of PD skin. In contrast, AK II predominantly displayed a chaotic/non-specific pattern, whereas AK III exhibited a mottled pattern. Subregions representing these lesion group-specific characteristics were selected for further analysis. Additional criteria included subregional image quality, defined by the presence of vessels throughout the entire subregion.

### Statistics

Descriptive statistics was performed for each vascular parameter calculated by OCTAVA and reported as mean and standard deviation (SD) for normally distributed data and as median with 25-75th interquartile range (IQR) for non-normally distributed data. Data were tested for normality using Kolmogorv-Smirnov normality test, evaluated using Tukey HSD corrected one-way ANOVA for normally distributed data and Kruskal-Wallis for non-normally distributed data, and visualized as boxplots. P-values were two-sided, exact, and considered statistically significant when < 0.05. Statistical analyses were performed using SPSS Statistics software (Version 28; IBM Corp., Armonk, New York, USA).

## Results

In this study, 45 patients (38 men and 7 women) with Fitzpatrick skin types I-III were enrolled. In field-cancerized skin of patient’s face (*n* = 166) or scalp (*n* = 39), a total of 205 AKs, including 93 AK I, 65 AK II, and 47 AK III, as well as 89 PD skin sites were scanned with D-OCT (Table 2). Subsequently, from these D-OCT scans, 18 scans of each clinical AK grade I-III and PD were selected for subregional assessment.

**Table 2:** Overview of D-OCT images from evaluated actinic keratosis (AK) and photodamaged (PD) skin on face or scalp of study participants

	PD	AK I	AK II	AK III	Total (*n*)
Number of lesions (n)	89	93	65	47	294
Face	69	72	50	44	235
Scalp	20	21	15	3	61
D-OCT scans (n)	89	93	65	47	294

PD; Photodamaged skin

AK; Actinic keratosis

**Fig. 1:**
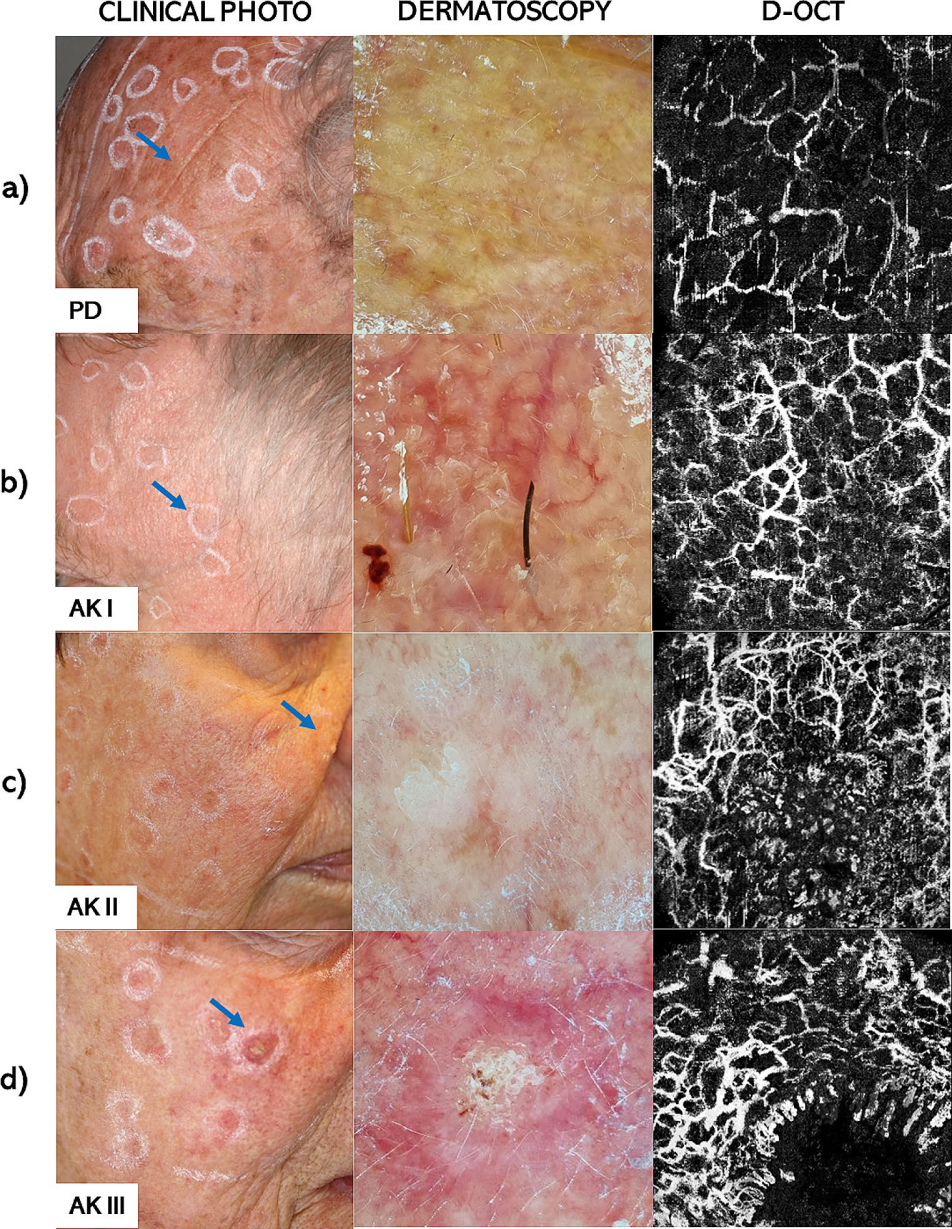
Clinical photographs, dermatoscopic images and en-face maximum intensity projections of D-OCT scans of (**a**) photodamaged (PD) skin and (**b**) actinic keratosis (AK) grades I, (**c**) AK II, and (**d**) AK III. Blue arrows mark the imaged lesions in the clinical photographs

**Fig. 2:**
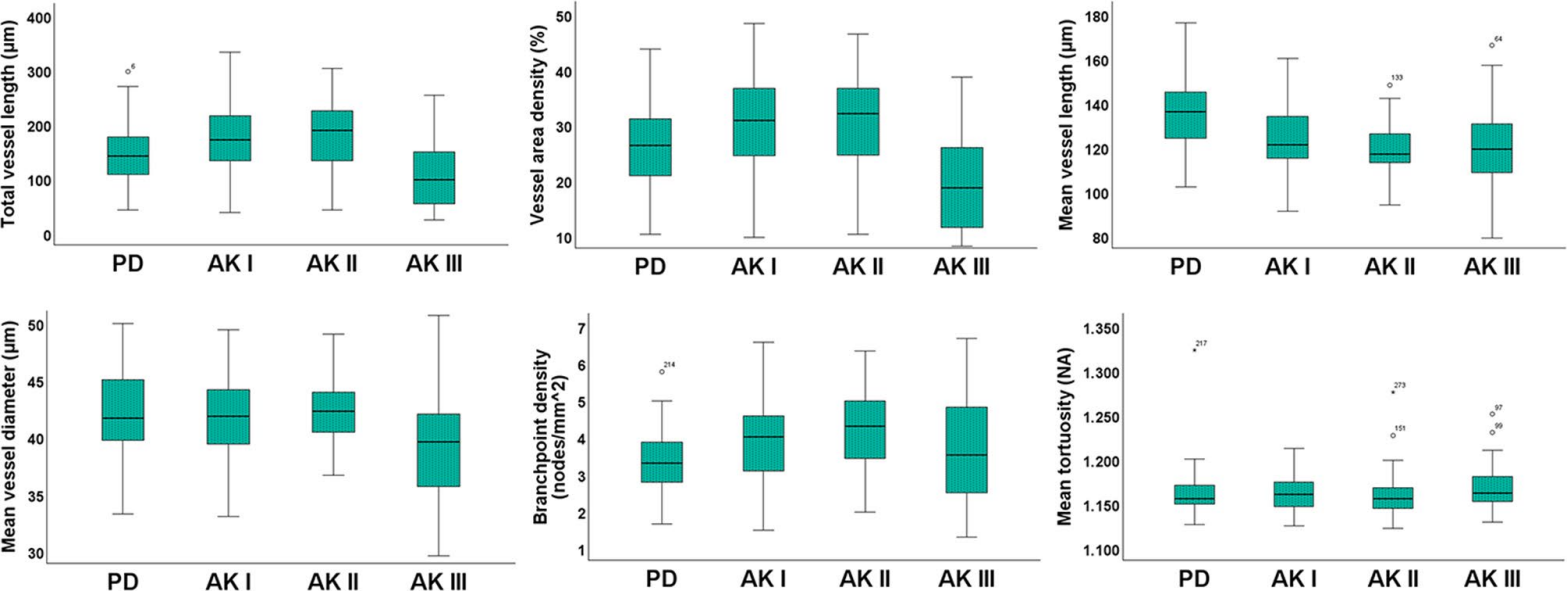
Boxplots of vascular parameters in actinic keratosis (AK) grades I (*n* = 93), II (*n* = 65), III (*n* = 47), and photodamaged skin (PD) (*n* = 89). For each parameter, the horizontal line in each box indicates the median value, boxes indicate the 25th and 75th percentile, while the whiskers represent the minimum and maximum values. Outliers are marked as individual points outside of the range of the whiskers

**Fig. 3:**
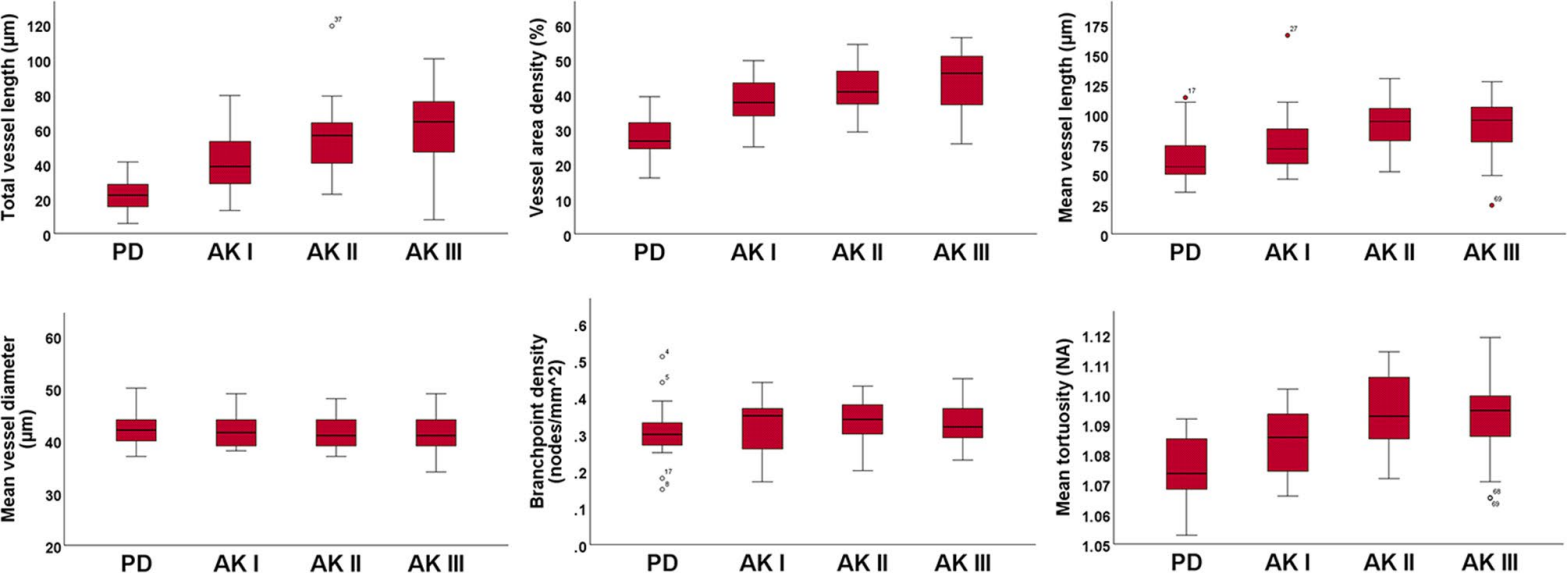
Boxplots of vascular parameters assessed in subregional analyses of actinic keratosis (AK) grades I (*n* = 18), II (*n* = 18), III (*n* = 18), and photodamaged skin (PD) (*n* = 18). For each parameter, the horizontal line in each box indicates the median value, boxes indicate the 25th and 75th percentile, while the whiskers represent the minimum and maximum values. Outliers are marked as individual points outside of the range of the whiskers

In our study, we combined D-OCT with OCTAVA to quantify microvascular features in AK I-III and PD skin. Our findings, illustrated in Figs. 1, 2 and 3, revealed significant differences in vascularization between AK and PD. A key finding was the association between the extent and disorganization of the vascular network across all AK grades compared to PD skin (Fig. 1). This association appeared in whole-regional assessments of AK I-II, as well as PD, as shown in Fig. 2. For AK III, subregional analysis was necessary to compensate for blood vessels obscured by hyperkeratosis. This approach confirmed the consistency of the observed trend in vascular alterations across all AK grades I-III, as depicted in Fig. 3. Furthermore, these subregional assessments highlighted distinct vascular differences among AK grades, with AK III displaying increased vascularization compared to AK I.

### Enhanced vascularization in AK

Vascular quantification revealed a pronounced increase in vascularization within all AK grades relative to PD (Table 3). This finding was supported by measuring total vessel length and VAD. In addition, measuring mean vessel length and diameter provided further insight into vascular network characteristics, despite inconsistent findings between whole-regional and subregional scans.

**Table 3:** Whole-regional OCTAVA assessments in actinic keratosis grades I-II and photodamaged skin

OCTAVA parameter (unit)	Lesion group	n	Mean	SD	p-value*
**Total length (µm)**	PD	89	145.456	50.1071	**< 0.001**
	AK I	93	170.921	60.0531	
	AK II	65	179.926	63.9329	
**Mean length (µm)**					
	PD	89	135.56	14.976	**< 0.001**
	AK I	93	123.12	13.976	
	AK II	65	118.45	13.249	
**Mean diameter (µm)**					
	PD	89	42,161	3,512	0.678
	AK I	93	41,795	3,527	
	AK II	65	42,203	2,685	
**Vessel area density (%)**					
	PD	89	26.326	7.124	**0.001**
	AK I	93	29.854	8.340	
	AK II	65	30.776	8.609	
**Branchpoint density (nodes/mm^2)**					
	PD	89	3,345	0.889	**< 0.001**
	AK I	93	3,933	1,072	
	AK II	65	4,268	1,068	
**Mean tortuosity (NA)**			**Median**	**IQR**	
	PD	89	1,559	1.149–1.171	0.625
	AK I	92	1,161	1.146–1.174	
	AK II	65	1,155	1.144–1.179	
**OCTAVA parameter (unit)**	**Post-hoc comparison**		p-value*		
**Total length (µm)**	AK I	PD	**0.009**		
	AK II	PD	**< 0.001**		
	AK II	AK I	0.600		
**Mean length (µm)**					
	AK I	PD	**< 0.001**		
	AK II	PD	**< 0.001**		
	AK II	AK I	0.105		
**Mean diameter (µm)**					
	AK I	PD	NA		
	AK II	PD			
	AK II	AK I			
**Vessel area density (%)**					
	AK I	PD	**0.009**		
	AK II	PD	**0.002**		
	AK II	AK I	0.756		
**Branchpoint density (nodes/mm^2)**					
	AK I	PD	**< 0.001**		
	AK II	PD	**< 0.001**		
	AK II	AK I	0.101		
**Mean tortuosity (NA)**					
	AK I	PD	NA		
	AK II	PD			
	AK II	AK I			

PD; Photodamaged skin

AK; Actinic keratosis

SD; Standard deviation

IQR; Interquartile range

NA; Not applicable

*Bold values indicate p ≤ 0.05

### Total vessel length

By quantifying vessel length, we found a difference in the extent of the vascular network between AK I-III and PD. As such, the total vessel length was significantly higher in AK I (*p* = 0.009) and AK II (*p* < 0.001) than in PD (Table 3). In addition, same trend persisted in AK III, as supported by subregional quantification that revealed a higher total length of vessels than in PD (*p* < 0.001) (Table 4).

**Table 4:** Subregional OCTAVA assessments in actinic keratosis grades I-III and photodamaged skin

OCTAVA parameter (unit)	Lesion group	n	Mean	SD	p-value*	
**Total length (µm)**	PD	18	23.342	1,09,167	**< 0.001**	
	AK I	18	41.268	2,03,732		
	AK II	18	54.588	2,31,936		
	AK III	18	60.877	2,50,930		
**Mean length (µm)**						
	PD	18	66.940	23,910	**0.014**	
	AK I	18	76.833	28,640		
	AK II	18	90.889	21,224		
	AK III	18	89.778	24,880		
**Mean diameter (µm)**						
	PD	18	42.556	3.518	0.810	
	AK I	18	41.889	3.234		
	AK II	18	41.778	3.318		
	AK III	18	41.444	3.838		
**Vessel area density (%)**						
	PD	18	28,357	6,867	**< 0.001**	
	AK I	18	38,081	7,007		
	AK II	18	41,567	7,355		
	AK III	18	44,214	8,986		
**Branchpoint density (nodes/mm^2)**						
	PD	18	0.397	0.084	0.696	
	AK I	18	0.323	0.076		
	AK II	18	0.333	0.062		
	AK III	18	0.330	0.062		
**Mean tortuosity (NA)**						
	PD	18	1,076	0.010	**< 0.001**	
	AK I	18	1,084	0.011		
	AK II	18	1,094	0.013		
	AK III	18	1,092	0.014		
**OCTAVA parameter (unit)**	**Post-hoc comparison**		**p-value**	**Post-hoc comparison**		p-value*
**Total length (µm)**						
	AK I	PD	0.053	AK II	AK I	0.222
	AK II	PD	**< 0.001**	AK III	AK I	**0.029**
	AK III	PD	**< 0.001**	AK III	AK II	0.797
**Mean length (µm)**						
	AK I	PD	0.632	AK II	AK I	0.332
	AK II	PD	**0.026**	AK III	AK I	0.405
	AK III	PD	**0.036**	AK III	AK II	0.999
**Mean diameter (µm)**						
	AK I	PD	NA	AK II	AK I	NA
	AK II	PD		AK III	AK I	
	AK III	PD		AK III	AK II	
**Vessel area density (%)**						
	AK I	PD	**0.002**	AK II	AK I	0.519
	AK II	PD	**< 0.001**	AK III	AK I	0.083
	AK III	PD	**< 0.001**	AK III	AK II	0.724
**Branchpoint density (nodes/mm^2)**						
	AK I	PD	NA	AK II	AK I	NA
	AK II	PD		AK III	AK I	
	AK III	PD		AK III	AK II	
**Mean tortuosity (NA)**						
	AK I	PD	0.186	AK II	AK I	0.089
	AK II	PD	**0.001**	AK III	AK I	0.196
	AK III	PD	**< 0.001**	AK III	AK II	0.981

PD; Photodamaged skin

AK; Actinic keratosis

SD; Standard deviation

NA; Not applicable

*Bold values indicate p ≤ 0.05

Moreover, subregional analysis identified an association between the extent of vascularization and AK thickness. Accordingly, our findings demonstrated a significant increase in total vessel length when comparing AK III to AK I (*p* = 0.029).

### Vessel area density

Further vessel quantification underscored a consistent trend of increased vascularization across all AK grades, when compared with PD. Specifically, VAD was higher in AK I-III than in PD. In AK I-II, this association was identified through whole-regional scan assessment, showing higher VAD in AK I (*p* = 0.009) and AK II (*p* < 0.002) than in PD. In AK III, we found an increased VAD compared with PD in subregional scans (*p* < 0.001).

### Mean vessel length and diameter

OCTAVA analyses revealed inconsistent trends of mean vessel length when comparing whole-regional and subregional D-OCT scans. While whole-regional analysis revealed a shorter mean length of vessels in AK I (*p* < 0.001) and II (*p* < 0.001) compared with PD, subregional assessment revealed a contrasting relationship (Tables 3 and 4). Thus, subregional vessel quantification showed a longer mean vessel length in AK II (*p* = 0.026) and AK III (*p* = 0.036) than in PD, while no difference was found between AK I and PD (*p* = 0.632).

Measurements of mean vessel diameter did not show any significant differences among AK I-III and PD, neither in whole-regional nor subregional scans (*p* ≤ 1.000).

### Disorganization within the vascular network in AK

Structural delineation of the vascular network unveiled distinct variations between AK I-III and PD. These variations manifested through both BD and mean tortuosity, as described below.

### Branchpoint density

In whole-regional scans, D-OCT displayed a significant increase in BD in AK I (*p* < 0.001) and II (*p* < 0.001) compared with PD (Fig. 2). Conversely, subregional assessments did not show any significant difference in BD, neither between AK I-III compared with PD nor between AK grades (Fig. 3).

### Mean tortuosity

An additional parameter that presented differently across whole-regional and subregional scans was mean tortuosity. Whole-regional analysis failed to detect any differences between AK and PD as well as among AK grades (Table 3). In contrast, subregional assessment identified significant differences between AK II-III and PD (Table 4). As such, in AK II (*p* = 0.001), and in AK III (*p* < 0.001), mean tortuosity was significantly higher compared with PD.

## Discussion

We characterized the microvasculature in clinical AK grades I-III and PD skin using D-OCT combined with OCTAVA for automated quantitative vessel analysis. Our study revealed, for the first time, vascular differences between each AK grade and PD skin, as well as among AK grades. Most notably, we observed a quantifiable association between the extent of vascularization and disorganization within the vascular network in all AK grades compared to PD skin. Subregional analysis supported these findings, allowing the identification of continuous vascular remodeling across AK I-III. Additionally, our subregional assessments demonstrated that as lesions thicken, vascularization increases, with thick AK III showing more extensive vascularization than thin AK I. Overall, these findings indicate D-OCT´s potential to supplement clinical AK grading by linking distinct subclinical alterations to surface features.

Prior D-OCT studies of keratinocyte cancer and its precursor lesions, including AK, have mostly relied on qualitative evaluation of images [7, 14–16]. These evaluations suffer from interobserver variability, which complicates comparisons across studies. Although a few published reports have quantitatively assessed vascular features in AK, they have typically focused on single parameters [15, 17]. These previous studies have described the presence of a reticular vascular network in AK with vessels of slightly larger diameter, and more superficially located than in healthy skin [7, 8, 15]. In contrast, our study´s more detailed approach using multiple quantitative parameters yielded more comprehensive insights into AK´s vascular network.

The clinical implication of vascular remodeling and increased thickness of AK remains unknown but is a significant finding considering that thin AK lesions respond more effectively to topical therapies, in contrast to thick, hyperkeratotic lesions [18–20]. In general, reduced treatment efficacy of thick AK is often attributed to restricted penetration of topical drugs or photosensitizers prior to PDT. Nonetheless, enhanced vascularization in thick AK could potentially also relate to their growth [21]. Thus, understanding this association might clarify why treating AK II-III presents more difficulties compared to AK I.

Implementation of non-invasive imaging tools to examine subclinical features of AK and field-cancerized skin may have potential to provide nuanced insights into disease management. Previous studies of AK have showed that the use of dermatoscopy and reflectance confocal microscopy after topical field-directed treatment can reveal persisting AK-associated changes in skin confirmed clear by clinical or histological examination [22, 23]. A gradual remission of AK-associated changes suggests that disappearance results from treatment induced epidermal degeneration and dermal remodeling, rather than from immediate physical destruction during treatment [22, 24]. Moreover, non-invasive monitoring post-treatment may be useful to detect early recurrence [25–28]. As such, D-OCT´s ability to assess dermal blood vessels may provide new opportunities to identify subclinical changes related to the effect of available therapeutic interventions.

Based on our results, whole-regional assessments provided a clearer distinction between AK I-II and PD than subregional scans. Many of the metrics calculated by OCTAVA are an average value over the scan area, so scanning over a larger area makes the measurement less sensitive to local heterogeneity which could cause more variability in the results. Another divergence between whole-regional and subregional assessments is the contrasting results of mean vessel lengths. This may be due to the fact that longer vessels are “cut off” when the image size is reduced, thereby artificially leading to a smaller mean vessel length. In whole-regional scans, the shorter mean vessel lengths observed in AK I-II relative to PD is more consistent with the presence of increased vascularization and disorganization of vessels in all AK grades visible qualitatively in the images. Therefore, it seems reasonable to reserve subregional assessments for situations where whole-regional imaging is hampered by particular skin structures.

Strengths of our study include the quantitated vessel assessments in-vivo. Compared to qualitative image interpretation, quantification of vessels enhances the reproducibility of our D-OCT findings. The automated analysis allowed the assessment of multiple parameters for microvascular characterization. Through subregional analysis, our study also addressed the challenge encountered in imaging hyperkeratotic AK III. This approach allowed comparison of variations in microvascular characteristics across different AK grades more precisely. Further, we applied multiple quantitative parameters to thoroughly characterize the vascular network, which offered a deeper understanding of more comprehensive insights into AK microvasculature compared with previous studies. Additionally, we included a large sample size of patients with AK of different clinical grades in the whole-regional analysis. Conversely, the reliability of our subregional vascular analysis may have been affected by the small sample size. This limitation underscores the importance of conducting corresponding analyses in a larger dataset to fully extend the applicability of these findings in a broader context. Also, future studies should assess the potential utility of assessing vascular features to predict treatment response.

## Conclusion

Automated quantification of multiple parameters for microvascular assessment unveiled subclinical vascular differences among AK grades I-III and PD skin. An increased vascularization and disorganization of the microvascular network characterizes all AK grades relative to PD skin. D-OCT with automated vascular analysis may serve as a supplement to clinical AK grading, potentially helpful to improve management strategies.

## Data Availability

No datasets were generated or analysed during the current study.
